# The Association Between Preoperative Patient-Reported Health Status and Postoperative Survey Completion Following Arthroplasty: Registry-Based Cohort Study

**DOI:** 10.2196/33414

**Published:** 2022-06-30

**Authors:** Ian A Harris, Yi Peng, Ilana Ackerman, Stephen E Graves

**Affiliations:** 1 Ingham Institute for Applied Medical Research School of Clinical Medicine University of New South Wales, Medicine and Health Liverpool Australia; 2 Australian Orthopaedic Association National Joint Replacement Registry South Australian Health and Medical Research Institute Adelaide Australia; 3 School of Public Health and Preventive Medicine Monash University Melbourne Australia

**Keywords:** total knee arthroplasty, total hip arthroplasty, patient-reported outcomes, perioperative medicine, postoperative medicine, knee surgery, arthroplasty, quality of life, surgical outcomes, cohort study, survey, health survey, hip, knee

## Abstract

**Background:**

Patient-reported outcome measures (PROMs) are commonly used to report outcomes after hip and knee arthroplasty, but response rates are rarely complete. Given that preoperative health status (as measured by PROMs) is a strong predictor of outcomes (using the same measures) and that these outcomes may influence the response rate, it is possible that postoperative response rates (the proportion of patients providing preoperative PROMs who also provide postoperative PROMs) may be influenced by preoperative health status.

**Objective:**

This study aims to test the association between preoperative PROMs and postoperative response status following hip and knee arthroplasty.

**Methods:**

Data from the PROMs program of the Australian national joint registry were used. The preoperative PROMs were the Oxford Hip Score or Oxford Knee Score, The EQ-5D Utility Index, and the EQ visual analog scale (VAS) for overall health. Logistic regression, adjusting for age, sex, BMI, and the American Society of Anesthesiologists (ASA) Physical Status Classification System, was used to test the association between each preoperative PROM and response status for the 6-month postsurgery survey.

**Results:**

Data from 9499 and 16,539 patients undergoing elective total hip arthroplasty (THA) and total knee arthroplasty (TKA) for osteoarthritis, respectively, were included in the analysis. Adjusting for age, sex, BMI, and ASA, there was no significant difference in response status at the postoperative follow-up based on the preoperative Oxford Hip or Knee Scores (odds ratio [OR] 1.00, 95% CI 0.99-1.01 for both; *P*=.70 for THA and *P*=.85 for TKA). Healthier patients (based on the EQ VAS scores) preoperatively were more likely to respond postoperatively, but this difference was negligible (OR 1.00, 95% CI 1.00-1.01 for THA and TKA; *P*=.004 for THA and *P*<.001 for TKA). The preoperative EQ Utility Index was not associated with the postoperative response rate for THA (OR 1.14, 95% CI 0.96-1.36; *P*=.13) or TKA patients (OR 1.05, 95% CI 0.91-1.22; *P*=.49).

**Conclusions:**

The likelihood of responding to a postoperative PROMs survey for patients undergoing hip or knee arthroplasty was not associated with clinically important differences in preoperative patient-reported joint pain, function, or health-related quality of life. This suggests that the assessment of postoperative outcomes in hip and knee arthroplasty is not biased by differences in preoperative health measures between responders and nonresponders.

## Introduction

Patient-reported outcome measures (PROMs) are commonly used to provide the patient’s perspective on outcomes such as pain, function, and quality of life after arthroplasty. However, response rates are rarely complete and vary between institutions and patients. If postoperative response rates are influenced by the preoperative severity of symptoms and quality of life, this would be a source of bias when estimating the average outcome from surgery because preoperative patient-rated pain, function, and quality of life are highly predictive of the corresponding postoperative outcomes, both short and long term [1-6]. Unfortunately, the outcome scores of nonresponders (by definition) cannot be measured, so it is not possible to know if there is bias in the response rate based on postoperative outcomes.

Measuring the association between preoperative PROMs scores and response status may provide insight into any potential postoperative responder bias. Evidence of responder bias would suggest that caution should be taken when interpreting average postarthroplasty PROMs from incomplete groups and that greater efforts to improve response rates may provide less biased results.

This study aims to determine if preoperative PROMs are associated with postoperative response status in patients undergoing elective hip or knee arthroplasty.

## Methods

### Overview

This retrospective cohort study uses a convenience sample of observational routinely collected data from the Australian Orthopaedic Association National Joint Replacement Registry (AOANJRR) PROMs program. The AOANJRR collects data on joint replacement surgery performed in all (over 300) hospitals in Australia performing arthroplasty surgery. The AOANJRR PROMs program was initiated in 43 hospitals in 2018 and data available from July 30, 2018, to January 38, 2020, were used for this study. Participating hospitals were chosen for the PROMs program to provide a cross-section of hospital types (including high and low volume, public and private, and metropolitan and regional) across Australian states and territories.

The study population included all patients undergoing elective total hip arthroplasty (THA) or elective total knee arthroplasty (TKA) for osteoarthritis at one of the 43 participating institutions who provided preoperative PROMs data. There were no exclusions. Data were collected directly from patients who entered their responses electronically (via smartphone, tablet, or computer) through the AOANJRR online data collection system. A telephone follow-up was performed for those who did not respond electronically. A more detailed description of the processes involved in the PROMs data collection for this cohort is provided elsewhere [7].

The primary outcome was response to the postoperative PROMs survey at 6 (minimum 5, maximum 8) months post surgery (yes or no) in patients who provided preoperative PROMs data. Predictor variables were preoperative PROMs scores: EQ-5D-5L Utility Index, EQ visual analog scale (VAS; an overall measure of quality of life from zero to 100 with 100 being the best possible health), and Oxford Hip/Knee Scores (joint-specific scores of pain and function from 0 to 48, with 48 being the best possible score). These PROMs were chosen for inclusion in the PROMs program by an international working group based on their common use among registries and in the clinical community, their validity and responsiveness to change in this population (arthroplasty), and their associated responder burden [7]. Using both PROMs provides a joint-specific profile (from the Oxford Score, allowing the collection of patient outcomes that are highly relevant to joint replacement and greater sensitivity to change from joint surgery) and a general health profile (from the EQ-5D scores, providing a better picture of overall health and allowing comparison with other health conditions).

### Ethics Approval

The following Australian ethics committees approved the pilot program from which these data were drawn: University of South Australia Human Research Ethics Committee (HREC; 200890), Sydney Local Health District Ethics Review Committee (RPAH Zone, HREC/18/RPAH/90), Calvary Health Care Adelaide HREC (18-CHREC-F004), Mater Misericordiae Ltd HREC (HREC/18/MHS/45), St Vincent’s Health and Aged Care HREC (HREC 18/14), University of Tasmania HREC (H0017292), Calvary Health Care Tasmania HREC (010418), St John of God HREC (1408), and Calvary Health Care (ACT; 25-2018). Consent was obtained for the collection and use of data, but consent was not obtained for the analyses used in this report as data were analyzed anonymously.

### Statistical Analysis

Data were analyzed descriptively, and logistic regression analyses adjusted for age, sex, American Society of Anesthesiologists (ASA) Physical Status Classification System score [8], and BMI were performed to test the association between each preoperative PROM score and response status. The association being tested is described visually in a directed acyclic graph (Multimedia Appendix 1). These covariates were chosen as they are key demographic and clinical variables routinely collected by the AOANJRR with a potential to impact the study outcome. A *P* value <.05 was considered statistically significant. Missing data were not imputed, as missingness was the dependent variable.

## Results

Data on 25,988 procedures were included in the analysis: 9449 THA and 16,539 TKA. The overall response rate post surgery for those who provided preoperative PROMs data was 82% (n=21,418), which varied from 41% (48/116) to 97% (208/215) between hospitals. For those who provided preoperative PROMs data, the distribution of patient characteristics based on response status for the postoperative survey is provided for THA and TKA in Tables 1 and 2, respectively. The proportion of female patients was between 3% and 4% higher for responders than nonresponders, for both THA and TKA. Differences between responders and nonresponders were less for other characteristics (sex, ASA, and BMI) except for healthier patients (ASA class 2 vs 3) being more likely to respond in the TKA group. The representativeness of responders versus nonresponders for this cohort has been previously reported [9].

The association between preoperative PROMs scores and postoperative response status, unadjusted and adjusted for age, sex, ASA score, and BMI, is provided for THA and TKA in Tables 3 and 4, respectively.

Patients undergoing THA or TKA who responded to the postoperative PROMs survey had significantly better preoperative scores for quality of life using the EQ VAS compared to nonresponders, but these differences were small (<2 points on a 100-point scale) and unlikely to be clinically important [10].

There was no significant association between the preoperative Oxford score or EQ-5D Utility Index and postoperative response status for patients undergoing THA or TKA (Tables 3 and 4). For the Oxford Scores, the differences were small (<1 point, smaller than a clinically important difference [11]) and the CIs were small.

**Table 1:** Summary of patient characteristics for those undergoing total hip arthroplasty who provided preoperative patient-reported outcome measures data by postoperative response status.

Variable			Did not respond (n=1752)		Responded (n=7697)		Total (N=9449)
Age (years), mean (SD)			65.8 (12.1)		66.2 (11.3)		66.1 (11.5)
**Sex, n (%)**							
	Female	883 (50.4)		4159 (54.0)		5042 (53.4)	
	Male	869 (49.6)		3538 (46.0)		4407 (46.6)	
**ASA^a,b^, n (%)**							
	1 (normal health)	160 (9.2)		558 (7.4)		718 (7.7)	
	2 (mild systemic disease)	949 (54.9)		4237 (56.0)		5186 (55.8)	
	3 (severe systemic disease)	600 (34.7)		2681 (35.4)		3281 (35.3)	
	4 (severe disease a threat to life)	21 (1.2)		89 (1.2)		110 (1.2)	
**BMI (kg/m^2^)^c^, n (%)**							
	Underweight (<18.50)	18 (1.1)		48 (0.7)		66 (0.8)	
	Normal (18.50-24.99)	341 (20.8)		1313 (19.7)		1654 (19.9)	
	Preobese (25.00-29.99)	597 (36.3)		2314 (34.6)		2911 (35.0)	
	Obese class 1 (30.00-34.99)	408 (24.8)		1773 (26.5)		2181 (26.2)	
	Obese class 2 (35.00-39.99)	178 (10.8)		823 (12.3)		1001 (12.0)	
	Obese class 3 (≥40.00)	101 (6.1)		410 (6.1)		511 (6.1)	

^a^ASA: American Society of Anesthesiologists.

^b^Excludes 154 procedures with unknown ASA score.

^c^Excludes 1125 procedures with unknown BMI.

**Table 2:** Summary of patient characteristics for those undergoing total knee arthroplasty who provided preoperative patient-reported outcome measures data by postoperative response status.

Variable		Did not respond (n=2818)	Responded (n=13,721)	Total (N=16,539)
Age (years), mean (SD)		67.9 (9.5)	67.7 (8.8)	67.7 (9.0)
**Sex, n (%)**				
	Female	1556 (55.2)	7998 (58.3)	9554 (57.8)
	Male	1262 (44.8)	5723 (41.7)	6985 (42.2)
**ASA^a,b^, n (%)**				
	1 (normal health)	122 (4.5)	623 (4.6)	745 (4.6)
	2 (mild systemic disease)	1425 (52.0)	7430 (55.3)	8855 (54.8)
	3 (severe systemic disease)	1167 (42.6)	5271 (39.2)	6438 (39.8)
	4 (severe disease a threat to life)	26 (0.9)	108 (0.8)	134 (0.8)
**BMI^c^(kg/m^2^), n (%)**				
	Underweight (<18.50)	3 (0.1)	12 (0.1)	15 (0.1)
	Normal (18.50-24.99)	274 (10.7)	1080 (9.3)	1354 (9.6)
	Preobese (25.00-29.99)	797 (31.2)	3301 (28.5)	4098 (29.0)
	Obese class 1 (30.00-34.99)	778 (30.4)	3553 (30.7)	4331 (30.7)
	Obese class 2 (35.00-39.99)	433 (16.9)	2088 (18.1)	2521 (17.9)
	Obese class 3 (≥40.00)	272 (10.6)	1531 (13.2)	1803 (12.8)

^a^ASA: American Society Anesthesiologists.

^b^Excludes 367 procedures with unknown ASA score.

^c^Excludes 2417 procedures with unknown BMI.

**Table 3:** Preoperative PROMs scores in patients undergoing total hip arthroplasty by response status post surgery.

PROMs^a^	Did not respond, mean (SD)	Responded, mean (SD)	Total, mean (SD)	Adjusted^b^ odds ratio (95% CI)	*P* value
EQ-5D-5L Utility	0.29 (0.37)	0.29 (0.37)	0.29 (0.37)	1.14 (0.96-1.36)	.13
EQ VAS^c^	63.7 (21.0)	65.2 (21.1)	65.0 (21.1)	1.00 (1.00-1.01)	.004
Oxford Hip Score	19.1 (8.7)	18.9 (9.0)	18.97 (8.99)	1.00 (0.99-1.01)	.70

^a^PROM: patient-reported outcome measure.

^b^Adjusted for age, sex, American Society Anesthesiologists score, and BMI; represents the likelihood of responding at 6 months.

^c^VAS: visual analog scale.

**Table 4:** Preoperative PROMs scores in patients undergoing total knee arthroplasty by response rate post surgery.

PROMs^a^	Did not respond, mean (SD)	Responded, mean (SD)	Total, mean (SD)	Adjusted^b^ odds ratio (95% CI)	*P* value
EQ-5D-5L Utility	0.39 (0.35)	0.40 (0.35)	0.39 (0.35)	1.05 (0.91-1.22)	.49
EQ VAS^c^	66.7 (19.8)	68.3 (19.5)	68.0 (19.6)	1.00 (1.00-1.01)	<.001
Oxford Knee Score	21.0 (8.7)	20.8 (8.4)	20.8 (8.5)	1.00 (0.99-1.01)	.85

^a^PROM: patient-reported outcome measure.

^b^Adjusted for age, sex, American Society Anesthesiologists score, and BMI; represents the likelihood of responding at 6 months.

^c^VAS: visual analog scale.

## Discussion

### Principal Results

For patients undergoing THA and TKA, there were no significant or clinically important differences in the preoperative Oxford scores or EQ Utility Index between those who responded to the postoperative survey and those who did not respond. There was evidence that patients with worse overall preoperative health on the EQ VAS for THA and TKA were less likely to respond, but these differences were small (<2-point difference on a 100-point scale for THA and <1-point difference for TKA, based on unadjusted data).

The findings suggest that arthroplasty patients who respond to PROMs surveys postoperatively are largely representative of patients who responded preoperatively regarding their preoperative PROMs. Similarly, the findings suggest that the postoperative outcomes data that are captured represent the full spectrum of patients regarding their capacity for improvement.

### Comparison With Prior Work

We have previously reported on the association between patient characteristics and response rates to PROMs surveys in this population, showing that postoperative responders are largely representative of the population undergoing surgery, with respect to age, sex, comorbidity, and BMI [11]. However, this study was not able to comment on the representativeness of preoperative PROMs data compared to all patients undergoing surgery, as data from nonresponders are not available. Similarly, we were unable to determine any association between postoperative PROMs and response rate.

Consistent with our findings, a study of Swedish Fracture Register participants found no significant differences in PROMs at baseline or at 1 year between responders and nonresponders [12]. However, *nonresponders* were patients who did not respond to the initial survey request but responded to a reminder. Therefore, patients who never responded were not included in the analysis.

In a registry study of total hip replacement recipients, nonresponders to follow-up surveys were found to have significantly worse EQ-5D and Oxford Hip Scores preoperatively [13]. The difference in baseline Oxford Hip Scores between responders and nonresponders at 6 months was 3 points, not a clinically important difference but larger than the difference found in this study. Similar findings were reported from the Swedish Knee Ligament Register, where responders to postoperative surveys were found to have better preoperative scores in two of the domains of the Knee Injury and Osteoarthritis Outcomes Score, but the differences were of questionable clinical importance [14].

A large UK study of postoperative survey response predictors at 6 months showed that better general health preoperatively (measured by the EQ Utility Index) was associated with increased probability of responding. This is consistent with our findings regarding general health using the EQ VAS [15].

### Limitations

The study findings should be interpreted in light of any limitations. The findings may not be generalizable to other countries or regions. The study findings may not be applicable to other periods of follow-up. The analysis was limited to available covariates, and the findings may be influenced by unmeasured confounders such as socioeconomic status. Furthermore, interaction from other variables (eg, patient characteristics) was not tested. However, the study used a large sample from a variety of hospitals, maximizing the power to detect any potential preoperative differences and the ability to generalize within Australia.

### Future Directions

Future studies assessing potential attrition bias in the reporting of patient-reported outcomes after surgery should include all likely confounders such as patient socioeconomic status, education, and specific comorbidities rather than the restrictive set of patient-level variables used in this study. Consideration should also be given to surgeon- and hospital-level variables when assessing factors that may influence response rates to postoperative surveys, although such data are rarely collected in a systematic manner.

### Conclusions

Patients responding to postoperative PROMs surveys following THA and TKA do not have clinically important differences in preoperative PROMs compared to those not responding. Preoperative scores are strong predictors of postoperative patient-reported outcomes, but this study suggests that the assessment of postoperative outcomes in hip and knee arthroplasty is not biased by differences in preoperative health measures between responders and nonresponders.

## Appendix

Multimedia Appendix 1 Directed acyclic graph for the association between preoperative patient-reported outcome measures and postoperative survey response.

## Abbreviations

AOANJRR: Australian Orthopaedic Association National Joint Replacement Registry
ASA: American Society of Anesthesiologists
HREC: Human Research Ethics Committee
OR: odds ratio
PROM: patient-reported outcome measure
THA: total hip arthroplasty
TKA: total knee arthroplasty
VAS: visual analog scale

## Data Availability

The Australian Orthopaedic Association National Joint Replacement Registry (AOANJRR) is declared by the Commonwealth of Australia as a federal quality assurance activity under section 124X of the Health Insurance Act, 1973. This declaration ensures freedom from subpoena and absolute confidentiality of information held by the registry. A declaration as a Quality Assurance Activity by the Commonwealth Minister of Health prohibits the disclosure of information that identifies individual patients or health care providers that is known solely as a result of the declared quality assurance activity.

External access to and use of deidentified AOANJRR data is permitted but must be in accordance with AOANJRR policies (Ref No POL.S3.3, S3.4, S3.5) available on the registry website [16]. Requests for data can be made by contacting the AOANJRR manager by email at admin@aoanjrr.org.au.
