# Supplementary figures and images for: The Association Between Preoperative Patient-Reported Health Status and Postoperative Survey Completion Following Arthroplasty: Registry-Based Cohort Study
Source: JMIR Perioper Med. 2022 Jun 30;5(1):e33414. doi: 10.2196/33414 (PMC9284355; doi:10.2196/33414)

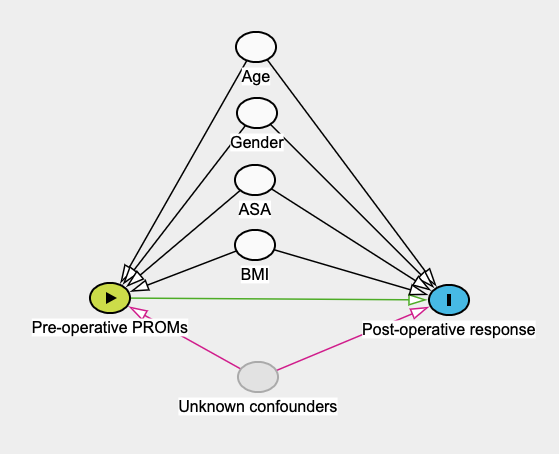

Supplement: Multimedia Appendix 1 [file periop_v5i1e33414_app1.png]
